# Supplementary material for: Exploring the molecular makeup of support cells in insect camera eyes
Source: BMC Genomics. 2023 Nov 22;24:702. doi: 10.1186/s12864-023-09804-5 (PMC10664524; doi:10.1186/s12864-023-09804-5)
Supplement: Supplementary file 1 — Additional file 1: SFig1. Expression of Cut in a subset of SupCs in the proximal portion of the eye tube. A. DAPI staining of eye tubes reveal nuclei in the periphery of eye tubes. B. A Cut antibody (green), labels clusters of SupC nuclei in the proximal portion of the eye tube. C. The overlay of DAPI and Cut staining illustrates that Cut positive nuclei comprise a sub-set of nuclei of SupCs. Scale bar = 100 µm. [file 12864_2023_9804_MOESM1_ESM.docx]

Supplementary Figure:


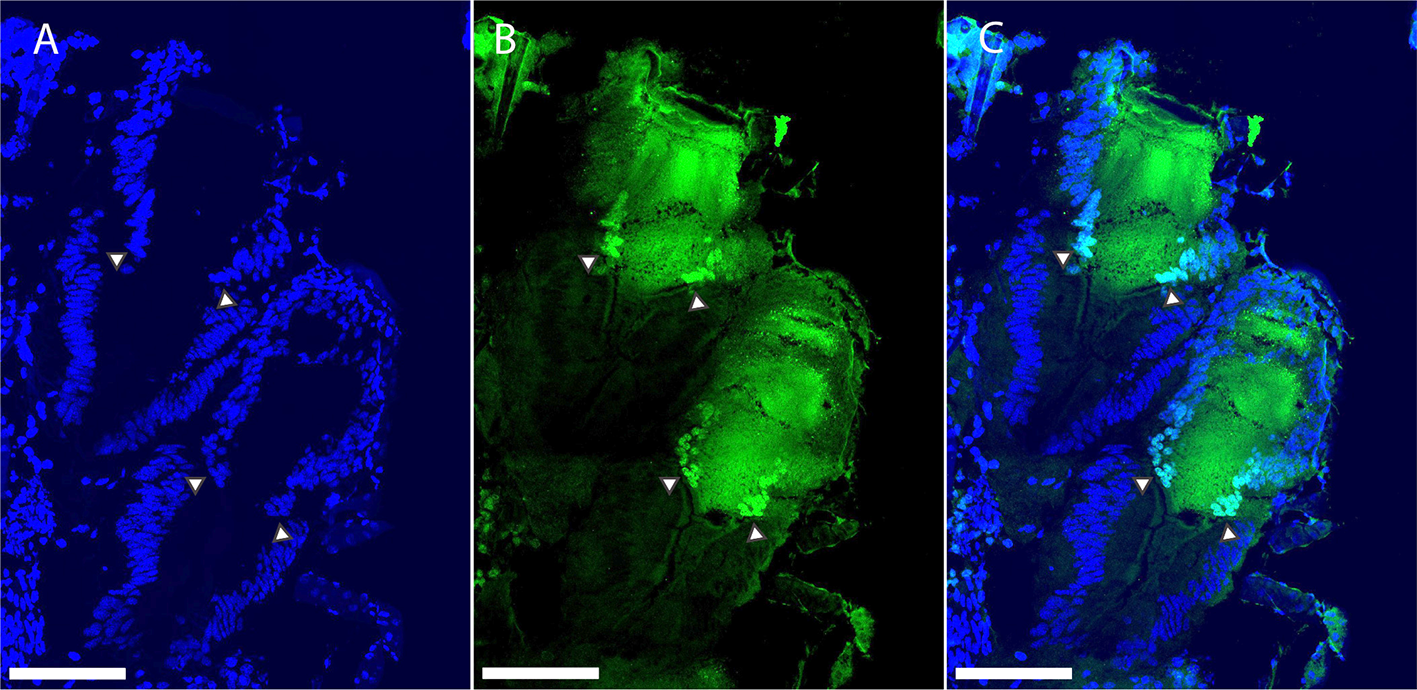


SFig1: Expression of Cut in a subset of SupCs in the proximal portion of the eye tube. A. DAPI staining of eye tubes reveal nuclei in the periphery of eye tubes. B. A Cut antibody (green), labels clusters of SupC nuclei in the proximal portion of the eye tube. C. The overlay of DAPI and Cut staining illustrates that Cut positive nuclei comprise a sub-set of nuclei of SupCs. Scale bar = 100 µm.
